# Systemic glucocorticoid exposure and postoperative infection risk in 143,782 appendectomy patients—a Danish longitudinal nationwide study

**DOI:** 10.1007/s00423-024-03294-z

**Published:** 2024-03-27

**Authors:** Doruk Orgun, Ask Tybjærg Nordestgaard, Henrik Enghusen Poulsen, Ismail Gogenur, Christina Ellervik

**Affiliations:** 1 Center for Surgical Science, Department of Surgery, Zealand University Hospital Køge and Roskilde, Køge, Denmark; 2 Department of Clinical Biochemistry, Copenhagen University Hospital - Herlev and Gentofte, Herlev, Denmark; 3 The Copenhagen General Population Study, Copenhagen University Hospital - Herlev and Gentofte, Herlev, Denmark; 4 Department of Clinical Medicine, Faculty of Health and Medical Sciences, University of Copenhagen, Copenhagen, Denmark; 5 Department of Clinical Pharmacology, Copenhagen University Hospital Bispebjerg and Frederiksberg, Copenhagen, Denmark; 6 Department of Cardiology and Endocrinology, Copenhagen University Hospital, Hillerød, Denmark; 7 Department of Pathology, Harvard Medical School, Boston, MA USA; 8 Department of Laboratory Medicine, Boston Children’s Hospital, Boston, MA USA; 9 Department of Clinical Biochemistry, Zealand University Hospital, Køge, Denmark

**Keywords:** Postoperative infections, Preoperative glucocorticoids, Laparoscopic appendectomy, Open appendectomy, Hypothalamic–pituitary–adrenal axis, Nationwide registry study

## Abstract

**Background:**

Glucocorticoids are conventionally associated with increased postoperative infection risk. It is necessary to clarify if preoperative glucocorticoid exposure is associated with postoperative infection in appendectomy patients and if the association is different for open and laparoscopic appendectomies.

**Methods:**

A Danish nationwide study of appendectomy patients between 1996 and 2018. Exposures were defined as high (≥ 5 mg) versus no/low (< 5 mg) glucocorticoid exposure in milligram prednisone-equivalents/day preoperatively. The main outcome was any postoperative infection. Then, 90-day cumulative incidences (absolute risk) and adjusted hazard ratios (relative risk) of the outcome were calculated for high versus no/low glucocorticoid exposure within all appendectomies and within open and laparoscopic subgroups. Propensity-score matching was used for sensitivity analysis.

**Results:**

Of 143,782 patients, median age was 29 years, 74,543 were female, and 7654 experienced at least one infection during the 90-day follow-up. The 90-day cumulative incidence for postoperative infection was 5.3% within the no/low glucocorticoid exposure group and 10.0% within the high glucocorticoid exposure group. Compared to no/low glucocorticoid exposure, adjusted hazard ratios for 90-day postoperative infection with high glucocorticoid exposure were 1.25 [95% CI 1.02–1.52; *p* = 0.03] for all appendectomies, 1.59 [1.16–2.18; *p* = 0.004] for laparoscopic appendectomies, and 1.09 [0.85–1.40; p = 0.52] for open appendectomies (*p*_interaction_ < 0.001). The results were robust to sensitivity analyses.

**Conclusion:**

Preoperative high (≥ 5 mg/day) glucocorticoid exposure was associated with increased absolute risk of postoperative infections in open and laparoscopic appendectomies. The relative risk increase was significant for laparoscopic but not open appendectomies, possibly due to lower absolute risk with no/low glucocorticoid exposure in the laparoscopic subgroup.

**Supplementary Information:**

The online version contains supplementary material available at 10.1007/s00423-024-03294-z.

## Introduction

Acute appendicitis is among the most common urgent surgical conditions worldwide with an incidence of 100–150 per 100,000 person-years in Europe and North America [1, 2]. Surgery is the standard treatment for acute appendicitis, and appendectomies are among the most frequently performed surgical procedures under general anesthesia [3, 4]. Laparoscopic appendectomies have been associated with lower incidences of postoperative surgical site infections compared with open appendectomies [5, 6].

Glucocorticoids are widely used for their anti-inflammatory properties in common chronic inflammatory conditions [7–11]. The prevalence of systemic glucocorticoid use is 1% in the UK and USA and 3% in Denmark [12–14]. Systemic glucocorticoid treatment using supraphysiological doses (≥ 5 mg prednisone-equivalents per day) is a common cause of secondary adrenal insufficiency due to suppression of the hypothalamic–pituitary–adrenal (HPA) axis [15]. The HPA axis is a central component of the neuroendocrine and immunological surgical stress response, and its suppression has been associated with an increased infection risk [16].

Previous studies on preoperative glucocorticoid exposure and postoperative infections have been restricted to elective procedures in which surgeons might recommend the tapering of glucocorticoids temporarily due to an anticipated higher risk of complications. In contrast, urgent surgical procedures such as appendectomy do not usually offer a setting where the preoperative tapering of glucocorticoids is an option due to the risk of adrenal crisis [17, 18]. Previous clinical studies examining associations between glucocorticoid treatment and postoperative infections in abdominal surgery are limited by their focus on specific patient groups (i.e., inflammatory bowel disease), by lack of detailed examination of dose–response relationships, and by their focus on selected infectious outcomes [19–23]. Furthermore, it is unknown whether a potential association depends on the type of procedure.

In this study, we tested the hypothesis that preoperative glucocorticoid exposure is associated with increased risk of postoperative infections within 90 days of urgent appendectomy. Including all urgent appendectomy patients in Denmark between 1996 and 2018, we calculated the 90-day cumulative incidence (absolute risk) and relative risk of postoperative infections for preoperative high (≥ 5 mg prednisone-equivalents per day) versus low (< 5 mg prednisone-equivalents per day) and no systemic glucocorticoid exposure, with particular focus on the open and laparoscopic appendectomy subgroups.

## Materials and methods

The study was approved by the Danish Data Protection Agency (Reference number: 2008–58-0028) and was conducted in accordance with the Declaration of Helsinki. The results are reported in compliance with the Strengthening the Reporting of Observational Studies in Epidemiology (STROBE) guidelines.

### Data sources

Information on surgical procedures including procedure codes, date of admission and discharge, and procedure type (open or laparoscopic) was collected from the Danish National Patient Registry. Information on diseases including diagnosis codes and dates was collected from the Danish National Patient Registry and the Danish National Causes of Death Registry. The Danish National Patient Registry [24] includes information on all hospital contacts (inpatient and outpatient clinics) in Denmark since 1977, and the Danish National Causes of Death Registry [25] includes information on the causes of all deaths in Denmark. Information on the type and dose of redeemed glucocorticoid prescriptions was collected using the Danish National Prescription Registry [26], which includes all redeemed prescriptions in Denmark since 1994. Data on age and gender were collected using The Danish Civil Registration System. Data from all registries were combined using the Danish personal identification number (CPR-number) that is unique for each individual.

### Population

We included all patients undergoing an appendectomy procedure in the Danish National Patient Registry (Nordic Classification of Surgical Procedures 2005 (NCSP 2005) codes: KJEA00, KJEA01, KJEA10) from January 1, 1996 (when emergency contacts were first included in the registries) to December 31, 2018 (last update of the registries). Patients undergoing surgery more than three days after hospital admission were excluded to ensure that only urgent procedures were included. Patients with a diagnosis of procedure-related infection (International Classification of Disease 2010 (ICD10) code: T81.4), pneumonia (J12–J18), acute cystitis (N30.0 and N30.9), acute pyelonephritis (N10), sepsis (A39.2, A40, A41, and T81.44), gas gangrene (A48.0), intestinal infectious disease (A00–A09), and generalized peritonitis or intestinal abscess unrelated to appendicitis (K63.0 and K65.0) at the time of admission were excluded. Additionally, patients with a previous diagnosis of Addison’s disease (E27.1), primary immune deficiencies (D80–D84 and D89), and hematological malignancies including myelodysplastic syndrome and polycythemia vera (C81–C96, D45, D46, and D47) at the time of admission were also excluded.

### Preoperative glucocorticoid exposure

We collected information on all redeemed glucocorticoid prescriptions on patients undergoing appendectomy from the Danish National Prescription Registry. Non-prednisone glucocorticoids were converted into prednisone-equivalent doses using previously published methods [27]. Assuming that redeemed prescriptions are adequate surrogates for actual glucocorticoid exposure, we calculated average daily glucocorticoid exposures as the cumulative amount of redeemed glucocorticoid prescriptions in milligram prednisone-equivalents for the 90 days preceding surgery divided by 90 for each patient. Then, we stratified the patients into groups of no/low glucocorticoid exposure (< 5 mg prednisone-equivalents per day) and high glucocorticoid exposure (≥ 5 mg/day). We chose 5 mg/day as cut off since exposures of ≥ 5 mg/day potentially suppress the HPA axis [15]. To examine dose–response relationships, patients were also stratified into groups of high (≥ 5 mg/day), low (> 0 and < 5 mg/day), and no glucocorticoid exposure (0 mg/day).

### Postoperative infections

A postoperative infection was defined as an inpatient diagnosis of infection following a procedure including surgical site infections (ICD10: T81.40–T81.43 and T81.49), pneumonia (J12–J18), acute cystitis (N30.0 and N30.9), acute pyelonephritis (N10), sepsis (A39.2, A40, A41, and T81.44), gas gangrene (A48.0), intestinal infectious diseases (A00–A09), acute peritonitis (K65.0), and intestinal abscess (K63.0). Infection diagnoses during the same admission and during readmissions within 90 days postoperatively were included.

### Co-variables

Co-variables included sex, age, year of surgery, procedure type (open or laparoscopic), complicated appendicitis, and patient comorbidities. Complicated appendicitis was defined as acute appendicitis with generalized or localized peritonitis (ICD10: K35.2 and K35.3) and gangrenous or phlegmonous appendicitis (K35.8B and K35.8C). Comorbidities included diabetes (ICD10: E10–E14), obstructive pulmonary diseases (J41–J46), inflammatory bowel diseases (K50 and K51), systemic autoimmune diseases and vasculitides (M02, M05–M09, M13.0, M30–M36, M45, M46.8, D59–D61, D69.0, D69.3, J82, J84, G35, G36.0, G61, E27.1, K74.3, K75.4, K83.01, K86.1, L10.0, L10.2, and L12), sarcoidosis (D86), and any non-hematological malignancy excluding in situ neoplasms and skin basal cell carcinoma (C00–C80). We further identified patients who had redeemed a prescription of an antidiabetic drug (ATC05 group: A10) as having diabetes to capture patients treated only in the primary sector.

### Statistical analyses

To compare differences between the distributions of baseline variables between the exposure groups at the time of surgery, we used the Mann–Whitney *U* test for continuous variables and Pearson’s chi-squared test for categorical variables. Two-sided *p* values less than 0.05 were considered statistically significant.

We calculated incidence rates per 1000 person-days as well as the 90-day cumulative incidences with corresponding 95% confidence intervals (95% CIs) for all postoperative infections among patients with high (≥ 5 mg prednisone-equivalents per day) and no/low glucocorticoid exposure (< 5 mg/day) in the entire cohort and in open and laparoscopic appendectomy subgroups. Corresponding Kaplan–Meier curves were also generated. Gray’s chi-squared test was used to compare the incidence rates. In addition, we calculated the total number of infection types and procedure types (open versus laparoscopic) in the entire cohort, as well as in patients with high (≥ 5 mg/day) versus no/low (< 5 mg/day) glucocorticoid exposure.

To compare postoperative infection risk in patients with high versus no/low glucocorticoid exposure, we calculated hazard ratios (HRs) for postoperative infections within 90 days postoperatively using unadjusted and multivariable Cox proportional hazards regression models in the entire cohort, in patients undergoing open surgery, and in patients undergoing laparoscopic surgery. The choice of 90 days as a cut-off was made to balance the need for specificity versus power. Adjustments included age, sex, procedure type (open versus laparoscopic), complicated appendicitis, diabetes, chronic obstructive pulmonary disease, inflammatory bowel disease, systemic autoimmune disease and vasculitis, sarcoidosis, and non-hematological malignancy. Additionally, we analyzed the interaction between procedure type (open or laparoscopic) and glucocorticoid exposure (none/low or high) by comparing regression models with and without interaction using minus-2 log likelihood test.

### Sensitivity analyses

To evaluate the robustness of our results, we used propensity-score subclassification matching for co-variable adjustment by using the same co-variables used in the multivariate Cox proportional hazards regression analyses. Propensity scores, which represent the estimated probability of exposure given patient baseline characteristics, were estimated by logistic regression for each individual, and then these individuals were assigned into subclasses. We then confirmed that all co-variables were balanced by matching propensity scores in the model (absolute standardized mean differences). Then, weights were computed for each subclass, and these were then used in the Cox model to estimate hazard ratios. We used the MatchIt package [28] in R to perform the propensity-score subclassification matching with the following specifications: “method = subclass” and “estimand = ATT”.

In order to compare postoperative infection risk within 90 days postoperatively between no glucocorticoid exposure versus low (> 0 mg/day and < 5 mg/day) and high exposure (≥ 5 mg/day) groups, we generated Kaplan–Meier curves as well HRs with corresponding 95% CIs for 90-day postoperative infections using unadjusted and multivariable Cox proportional hazards regression models. We adjusted for the same co-variables as in the main analyses.

Since we could not differentiate whether the infection diagnoses that were registered on the day of admission were already present at admission or occurred postoperatively, we performed sensitivity analyses without excluding patients with an infection diagnosis on the day of admission. In addition, mixed-effect Cox regression analyses were performed to account for the clustering of patients at hospital level. Also, to assess confounding by indication, we additionally performed stratified analyses for the major disease groups where glucocorticoids are indicated. Finally, to explore whether the impact of the predictor effect of glucocorticoid exposure on the outcome was mediated by “complicated appendicitis,” we excluded this co-variable in an additional sensitivity analysis.

All statistical analyses were performed using the Statistics Denmark server via a remote desktop application and using the R programming language version 4.2.1 (R Core Team (2020). R: A language and environment for statistical computing. R Foundation for Statistical Computing, Vienna, Austria. URL: https://www.R-project.org/).

## Results

**Fig. 1:**
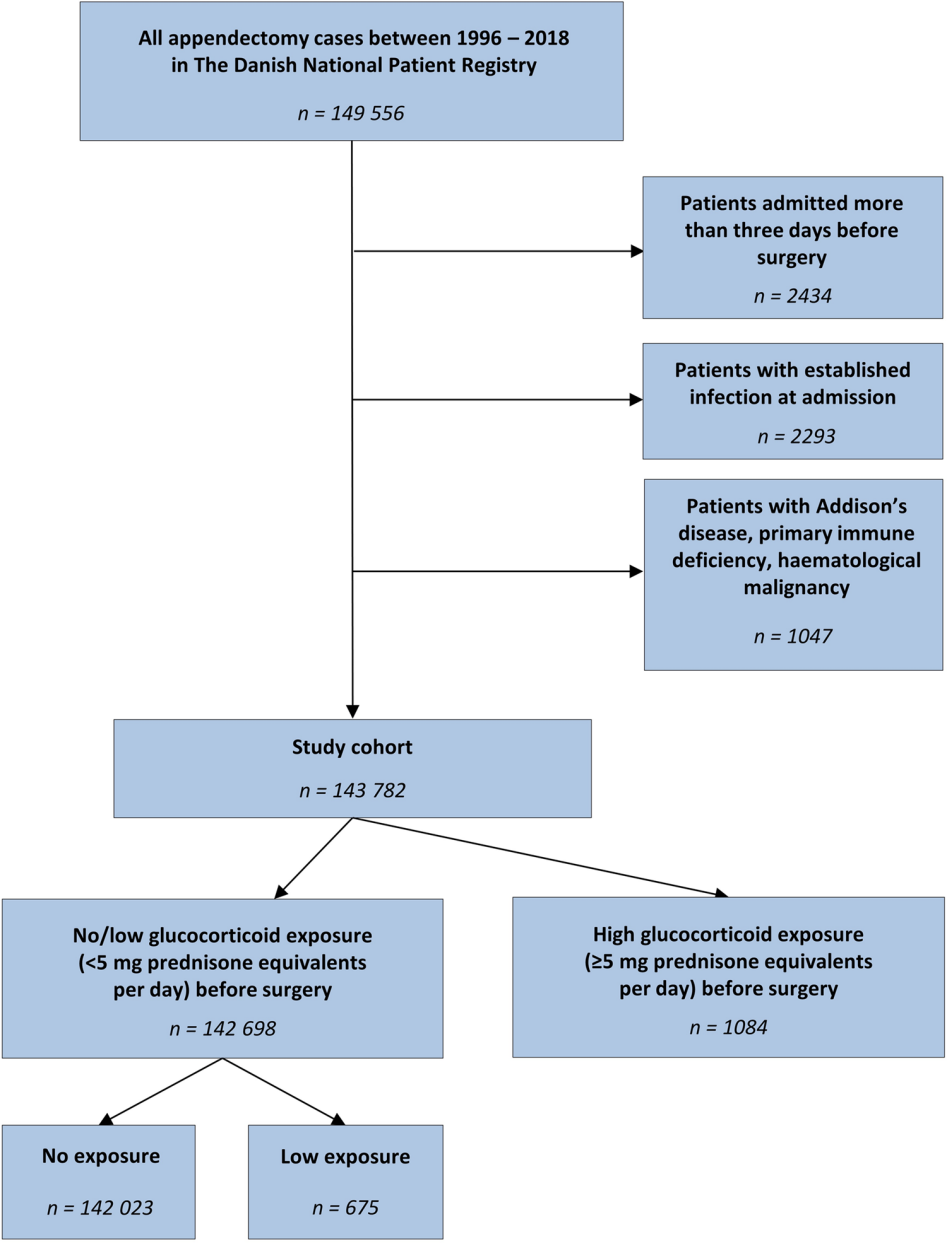
Flowchart of patients undergoing appendectomy in Denmark from 1996 to 2018. Low glucocorticoid exposure is defined as > 0 and < 5 mg prednisone equivalents per day and high glucocorticoid exposure as ≥ 5 mg prednisone equivalents per day

From 1996 to 2018, we identified 149,556 patients undergoing appendectomy in the Danish National Patient Registry. We excluded 2434 patients who were admitted more than three days before the date of surgery, as well as 2293 patients who were diagnosed with a procedure-related infection, pneumonia, acute cystitis, acute pyelonephritis, sepsis, gas gangrene, intestinal infectious disease, and generalized peritonitis or intestinal abscess unrelated to appendicitis at the time of admission. Then, 1047 patients with a previous diagnosis of Addison’s disease, primary immune deficiency, hematological malignancy including myelodysplastic syndrome, and polycythemia vera were excluded, leaving 143,782 patients for further analyses **(**Fig. 1**)**.

Median age was 29 years (interquartile range 17–49), and 74,543 patients (51.8%) were female. Median length of hospital stay was 2 days (interquartile range 1–4), and 76,913 patients (53.5%) underwent open appendectomy. Then, 35,276 patients (24.5%) were diagnosed with complicated appendicitis. Among the included 143,782 patients, 142,023 (98.8%) were not exposed to glucocorticoids (no exposure), 675 (0.5%) received glucocorticoids with an average daily prednisone-equivalent glucocorticoid dose of > 0 and < 5 mg/day (low exposure), and 1084 (0.8%) received a dose of ≥ 5 mg/day (high exposure) within 90 days preoperatively **(**Table 1, Supplementary Table 1**)**.

**Table 1:** Baseline characteristics of patients undergoing appendectomy in Denmark from 1996 to 2018 according to glucocorticoid exposure

	No glucocorticoid exposure (*n* = 142,023)	Low glucocorticoid exposure* (*n* = 675)	High glucocorticoid exposure* (*n* = 1084)	Total (*n* = 143,782)
Age, years (IQR**)	29 (16–49)	49 (31–66)	64 (42–74)	29 (17–49)
Preoperative glucocorticoid exposure				
Median exposure, mg/day (range)	0	2.50 (0.25–4.99)	9.26 (5–250)	0 (0–250)
Sex, *n* (%)				
Female	73,485 (51.7)	404 (59.9)	654 (60.3)	74,543 (51.8)
Length of stay, days (IQR**)	2 (1–4)	3 (1–5)	4 (2–7)	2 (1–4)
Type of surgery, *n* (%)				
Open	75,889 (53.4)	377 (55.9)	647 (59.7)	76,913 (53.5)
Laparoscopic	66,134 (46.6)	298 (44.1)	437 (40.3)	66,869 (46.5)
Complicated appendicitis, *n* (%)	34,886 (24.6)	153 (22.7)	237 (21.9)	35,276 (24.5)
Comorbidities, *n* (%)				
Diabetes	2892 (2.0)	22 (3.3)	79 (7.3)	2993 (2.1)
Malignancy	8805 (6.2)	72 (10.7)	205 (18.9)	9082 (6.3)
Autoimmune disease	3844 (2.7)	89 (13.2)	318 (29.3)	4251 (3.0)
Inflammatory bowel disease	1646 (1.2)	25 (3.7)	105 (9.7)	1776 (1.2)
Obstructive pulmonary disease	8373 (5.9)	116 (17.2)	297 (27.4)	8786 (6.1)

^*^Low glucocorticoid exposure: > 0 mg and < 5 mg prednisone equivalents per day, high glucocorticoid exposure: ≥ 5 mg prednisone equivalents per day. **IQR, interquartile range

**Fig. 2:**
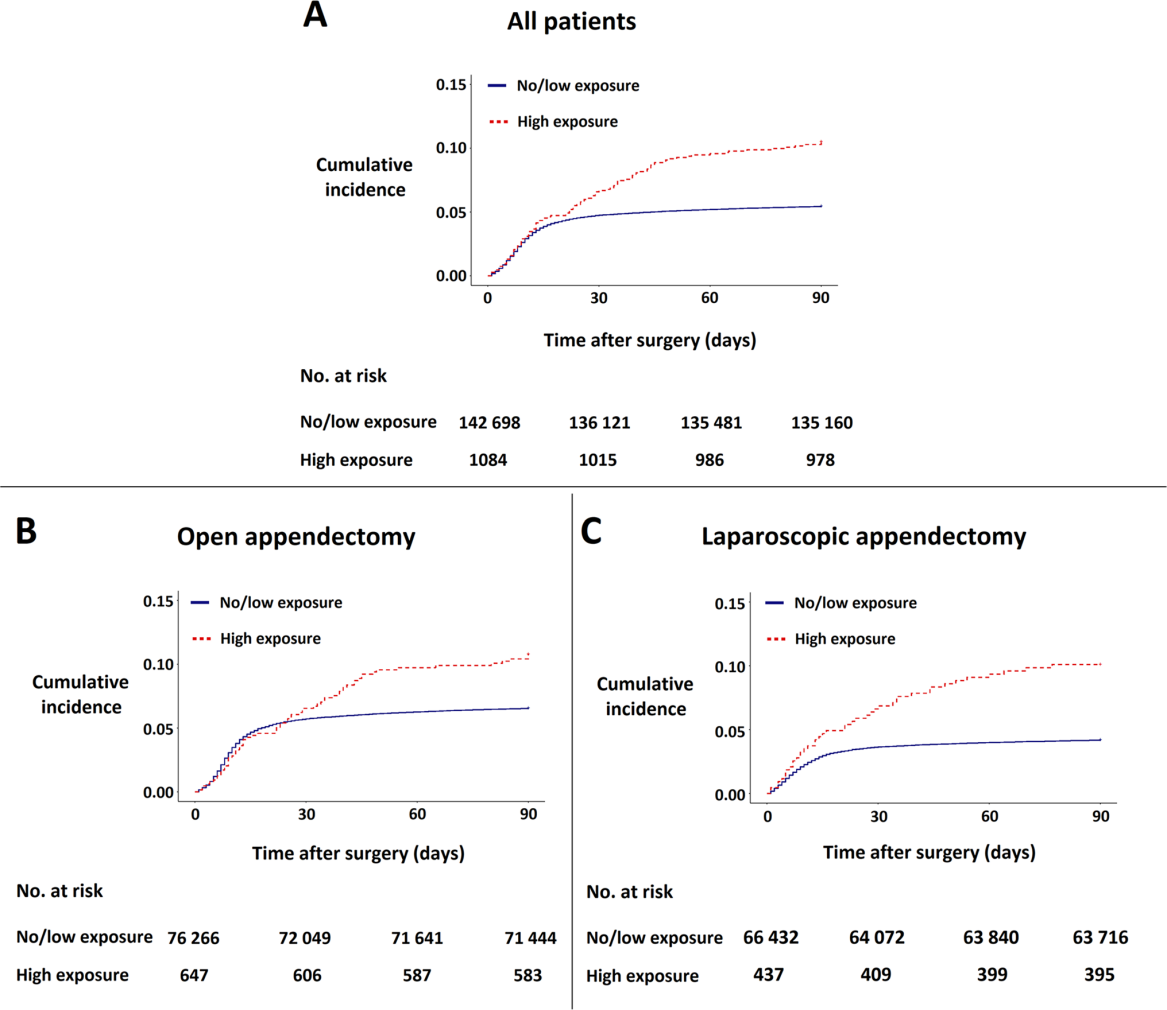
Kaplan–Meier curves for the 90-day cumulative incidence of postoperative infections in all appendectomies **(A)**, in open appendectomies **(B)**, and in laparoscopic appendectomies **(C)**. Patients were stratified into groups of no/low glucocorticoid exposure (< 5 mg prednisone equivalents per day) and high glucocorticoid exposure (≥ 5 mg prednisone equivalents per day). Log-rank test *p* values for differences between groups in **A**, **B**, and **C**: < 0.001

A total of 7654 patients were diagnosed with at least one postoperative infection within 90 days of surgery, 108 in the high exposure (≥ 5 mg/day) group, and 7546 in the no/low exposure (< 5 mg/day) group (*p* < 0.001) **(**Table 2**)**. In the no/low exposure group, 72% of the postoperative infections occurred during the first two weeks (postoperative days 0–15) and were dominated by SSI and intraabdominal (32%) and UTI and other infections (33%). In the high-exposure group, 48% of the postoperative infections occurred during the first two weeks and were more equally distributed among the infection subtypes (Supplementary Table 2). The 90-day cumulative incidence for postoperative infection was 5.3% (95% CI 5.2–5.4) within the no/low exposure group and 10.0% (8.3–11.9) within the high exposure group. Corresponding estimates for open and laparoscopic appendectomy subgroups are presented in Table 2, and the corresponding Kaplan–Meier curves for no/low exposure and high exposure in all appendectomies as well as the open and laparoscopic appendectomy subgroups are presented in Fig. 2. Then, 90-day cumulative incidences of infection for no/low exposure and high exposure groups were found to be significantly different (*p* < 0.001).

**Table 2:** Incidence rates of postoperative infections, infection subtypes, and procedure types according to glucocorticoid treatment groups in patients undergoing appendectomy in Denmark from 1996 to 2018

	No/low glucocorticoid exposure* (*n* = 142,698)	High glucocorticoid exposure* (*n* = 1084)	Total (*n* = 143,782)	*p* value
≥ 1 infections, *n*	7546	108	7654	< 0.001
Open surgery	4827	66	4893	< 0.001
Laparoscopic surgery	2719	42	2761	< 0.001
Incidence, 90-day cumulative (95% CI)				
All	5.3% (5.2–5.4)	10.0% (8.3–11.9)	5.3% (5.2–5.4)	< 0.001
Open surgery	6.3% (6.2–6.5)	10.2% (8.1–12.8)	6.4% (6.2–6.5)	< 0.001
Laparoscopic surgery	4.1% (3.9–4.2)	9.6% (7.2–12.8)	4.1% (4.0–4.3)	< 0.001
Incidence rate, per 1000 person-days (95% CI)				
All	0.61 (0.60–0.63)	1.19 (0.98–1.44)	0.61 (0.60–0.63)	< 0.001
Open surgery	0.74 (0.72–0.76)	1.22 (0.94–1.55)	0.75 (0.73–0.77)	< 0.001
Laparoscopic surgery	0.47 (0.45–0.49)	1.15 (0.83–1.55)	0.47 (0.46–0.49)	< 0.001
Infection type, *n* (%)				
Surgical site infection	1061 (0.7)	10 (0.9)	1071 (0.7)	0.61
Intraabdominal	2350 (1.6)	30 (2.8)	2380 (1.7)	0.006
Pneumonia	717 (0.5)	33 (3.0)	750 (0.5)	< 0.001
Urinary tract	692 (0.5)	11 (1.0)	703 (0.5)	0.02
Sepsis	354 (0.2)	13 (1.2)	367 (0.3)	< 0.001
Other infections	3076 (2.2)	30 (2.8)	3106 (2.2)	0.20

^*^No/low glucocorticoid exposure: < 5 mg prednisone equivalents per day, high glucocorticoid exposure: ≥ 5 mg prednisone equivalents per day. *CI*, confidence interval

**Fig. 3:**
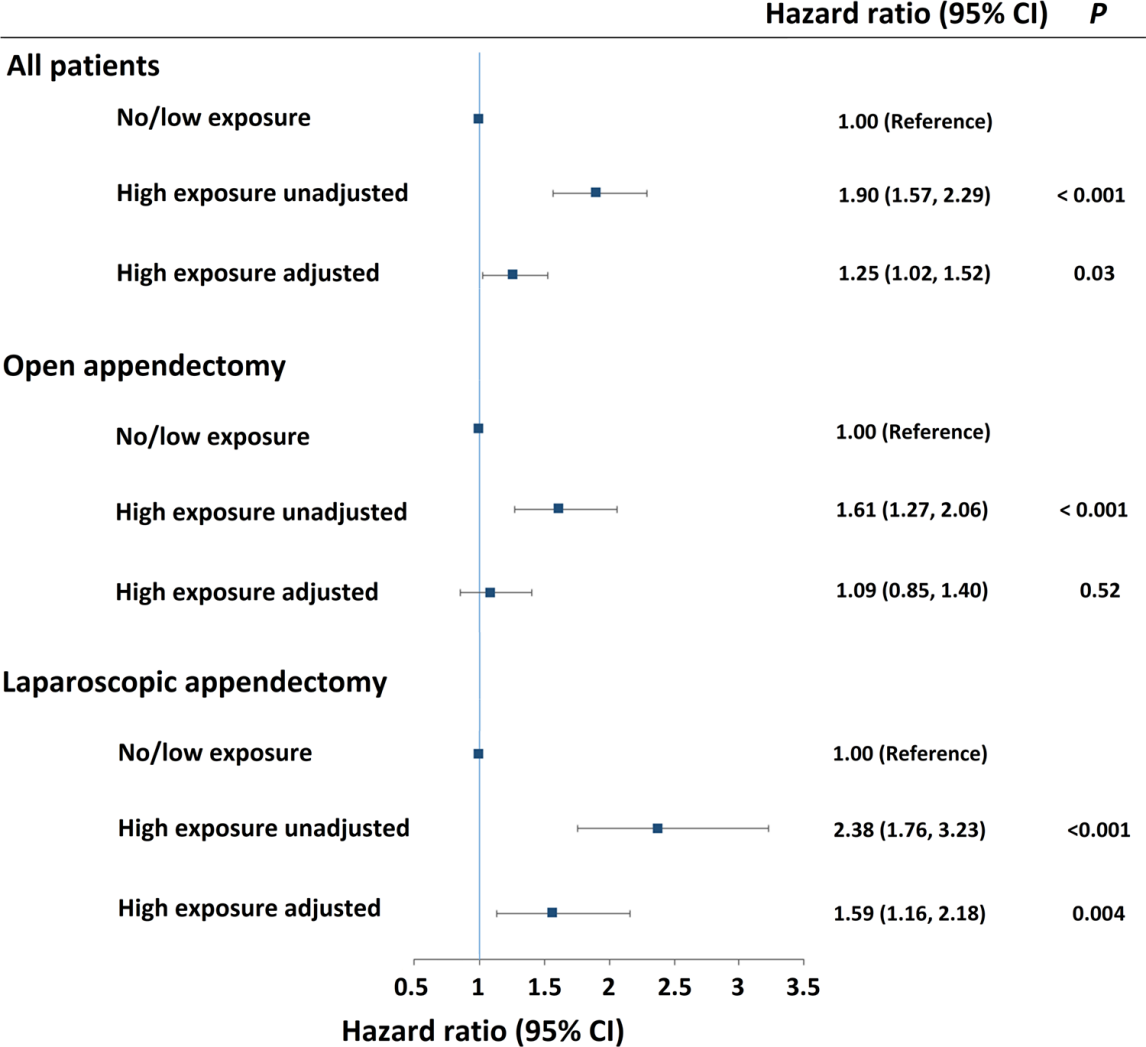
Hazard ratios for 90-day postoperative infections in all appendectomies, in open appendectomies, and in laparoscopic appendectomies. Patients were stratified into groups of no/low glucocorticoid exposure (< 5 mg prednisone equivalents per day) and high glucocorticoid exposure (≥ 5 mg prednisone equivalents per day). The hazard ratios were calculated using unadjusted and multivariate Cox proportional hazard regressions adjusted for age, sex, procedure type, and comorbidities. Analyses included 143,782 patients undergoing appendectomy. CI, confidence interval

Using Cox proportional hazard regression, the multivariable-adjusted HRs for postoperative infections in patients with high exposure (≥ 5 mg/day) versus no/low exposure (< 5 mg/day) were 1.25 (1.02–1.52; *p* = 0.03) for all appendectomies, 1.59 (1.16–2.18; *p* = 0.004) for the laparoscopic appendectomy subgroup, and 1.09 (0.85–1.40; *p* = 0.52) for the open appendectomy subgroup (*p*_interaction_ < 0.001) **(**Fig. 3**)**.

### Sensitivity analyses

We used propensity scores to adjust the absolute standardized mean differences of all aforementioned covariates to be less than the threshold of 0.05 and confirmed that there was an acceptable covariate balance (Supplementary Fig. 1). During propensity-score matching, we avoided the exclusion of individuals by using a subclassification matching method. Using the weights from the matching, adjusted hazard ratio for postoperative infections among the high exposure (≥ 5 mg/day) group compared with the no/low exposure (< 5 mg/day) group was 1.23 (95% CI 1.08–1.41; *p* = 0.002) for all appendectomies. Corresponding hazard ratio estimates for the open and laparoscopic appendectomy subgroups are presented in Supplementary Fig. 2.

In addition, the multivariable-adjusted hazard ratios for postoperative infections within all appendectomies were 0.74 (0.52–1.04; *p* = 0.08) for low exposure (< 5 mg/day) and 1.24 (1.02–1.51; p = 0.03) for high exposure (≥ 5 mg/day) when comparing with no exposure as baseline (Supplementary Fig. 3). Corresponding Kaplan–Meier curves are shown in Supplementary Fig. 4.

Sensitivity analyses for postoperative infections in patients with high exposure (≥ 5 mg/day) versus no/low exposure (< 5 mg/day) (a) without excluding patients with an infection diagnosis on the day of admission, (b) with mixed effect Cox regression to account for the clustering of patients at hospital level, (c) by the stratification of major disease groups where glucocorticoids are indicated, (d) by excluding the “complicated appendicitis” co-variable, all showed similar results (Supplementary Table 3).

## Discussion

In this large Danish nationwide study, we have demonstrated that while the absolute risk of postoperative infections increased with high glucocorticoid exposure both in open and laparoscopic appendectomies, the relative risk increase attributed to high glucocorticoid exposure was significantly higher only for laparoscopic appendectomies. This was thought to be the result of a lower absolute risk in the no/low glucocorticoid exposure group within laparoscopic appendectomies. Postoperative infections in the no/low glucocorticoid exposure group occurred mainly during the first two weeks, whereas in the high glucocorticoid exposure group, infections occurred up to 60 days postoperatively. Surgical site and intraabdominal infections as well as urinary tract and other infections were more prevalent in the early postoperative phase for the no/low glucocorticoid exposure group, whereas infections were more equally distributed throughout the follow-up period in the high glucocorticoid exposure group. These results are novel. In addition, the risk of postoperative infections was lower among laparoscopic appendectomy patients compared with open appendectomy patients, which is in line with previous studies [29]. The results were robust to various sensitivity analyses.

Sensitivity analyses further suggested that the exposure of 5 mg prednisone equivalent glucocorticoids per day might be a biological threshold since exposure to lower amounts of glucocorticoids was not found to have associations with increased risk of postoperative infections. Total daily dosages that exceed physiologic equivalents (≥ 5 mg prednisone/prednisolone/day) are associated with secondary adrenal insufficiency and hypothalamic–pituitary–adrenal axis dysfunction, which results in the decrease of physiological steroid hormone secretion from the adrenal cortex [15].

Systemic glucocorticoid use is intended for immunosuppression but is associated with a general adverse infection risk [30]. In surgical patients, systemic glucocorticoid use has traditionally been associated with impaired wound healing and surgical site infections, especially with high dose or long-term treatment [31]. Comparing results from studies on glucocorticoid treatment and adverse postoperative outcomes is challenging due to the lack of consensus in the definition of high dose/chronic/long-term glucocorticoid exposure and the inadequate listing of exact treatment doses and duration in most studies [32–34]. For example, in a large observational study of more than 600,000 patients, glucocorticoid treatment within 30 days before surgery was associated with five times higher risk of wound infections, although the dose and duration of glucocorticoid treatment as well as the procedure types were not specified [23]. Furthermore, other studies investigating the association between glucocorticoids and risk of postoperative infections have mostly focused on patients with one particular disease for which glucocorticoid treatment is indicated. In a large study (*n* = 15,495) of patients with inflammatory bowel disease undergoing major abdominal surgery, the odds ratios (ORs) for postoperative infections with preoperative glucocorticoid use were 1.22 (95% CI 1.06–1.39) for Crohn’s disease and 1.30 (95% CI 1.14–1.49) for ulcerative colitis patients, albeit treatment length and doses were unspecified [35]. In another study (*n* = 159) of patients with inflammatory bowel disease undergoing elective bowel surgery, the OR for postoperative infections was 3.69 (95% CI 1.24–10.97) with glucocorticoid treatment (median dose of 20 mg prednisone/day) versus untreated patients. Importantly, the regression models in this study were not adjusted for disease-specific variables and patient comorbidities, and preoperative glucocorticoid dose calculations were not described in detail [36]. Finally, in a large observational study (*n* = 10,777) of patients with rheumatoid arthritis undergoing hip, cardiac, or abdominopelvic surgery, preoperative glucocorticoid treatment of ≥ 5 mg prednisone per day versus < 5 mg/day was associated with high risk of wound complications including infections (OR for 5–10 mg/day: 1.72 [1.24–2.39]; OR for > 10 mg/day: 1.68 [0.97–2.94]) [19].

Postoperative antibiotic regimens for patients undergoing appendectomy are highly debated. In a recent multicenter randomized controlled trial, postoperative two-day intravenous antibiotic regimen in patients with complicated appendicitis undergoing laparoscopic appendectomy was non-inferior to a five-day regimen; however, patients with autoimmune diseases or immunosuppressant therapy, including glucocorticoid treatment, were excluded [37–39]. Therefore, it is still unknown if shorter-term postoperative antibiotic treatment would benefit laparoscopic appendectomy patients with preoperative glucocorticoid exposure. Although this trial only investigated patients with complicated appendicitis, our results suggest that particularly for laparoscopic appendectomy patients with ≥ 5 mg/day glucocorticoid exposure, surgeons should be aware of and inform patients about the association of increased risk for postoperative infections.

### Strengths and limitations

The strengths of this study include a large population undergoing the same type of surgical procedure. We included all appendectomy patients from 1996 to 2018 in Denmark including all ages and ethnic backgrounds. Further, in contrast to most previous studies on glucocorticoid treatment and adverse postoperative outcomes, we have used a clear and precise method to define groups of glucocorticoid treatment. The appendectomy procedure codes in the Danish National Patient Registry have been shown to be highly accurate [40, 41]; thus, the risk of misclassification for the procedure is low; however, the Danish National Patient Registry does not hold information on which laparoscopic procedures were converted to open procedures intraoperatively. Also, although Danish National Patient Registry includes “complicated appendicitis” with its corresponding diagnosis codes such as “appendicitis with generalized peritonitis,” there are no specific data on “perforation” as a cause to the complicated nature of these diagnoses in our dataset. Regarding the outcomes, we cannot rule out any possible misclassification of infection diagnoses in the Danish National Patient Registry; however, the registry is shown to be of high validity [24]. Additionally, we did not have information on infections only treated in the primary sector (not leading to a hospital contact); therefore, it is likely that we did not capture minor infections. Moreover, glucocorticoid exposure groups were based on the number of redeemed glucocorticoid prescriptions, and although the Danish National Prescription Registry is very precise by including all prescribed and redeemed prescriptions in the entire country, the details of the actual prescribed treatment protocol and patient compliance are not available [26]. Although there was no missing data regarding baseline characteristics, patient comorbidities, and glucocorticoid exposure, we cannot exclude if some of these data were not adequately captured. In addition, to maximize power, we chose to include all patients undergoing appendectomy rather than a pre-specified patient group (e.g., rheumatoid arthritis). It is possible that our results are partly explained by confounding the disease for which the glucocorticoid treatment was initially initiated, although we included adjustment for comorbidities. Also, it is possible that the level of glucocorticoid treatment is a marker of inflammatory disease severity. Finally, since this is an observational study, the presence of residual confounding is inevitable.

## Conclusions

In 143,782 patients from the Danish population undergoing appendectomy, high glucocorticoid exposure was associated with an increased absolute risk of 90-day postoperative infections in both open and laparoscopic appendectomies. However, the relative risk increase was significant only for laparoscopic appendectomies with a 59% increase, possibly due to a lower absolute risk with no/low glucocorticoid exposure within this subgroup. For these patients, exposure to preoperative glucocorticoids should be taken into consideration when planning postoperative care, regardless of whether they have undergone a common minimally invasive surgical procedure.

### Supplementary Information

Below is the link to the electronic supplementary material.

Supplementary file1 (DOCX 860 KB)

## Data Availability

No datasets were generated or analysed during the current study.
