# Supplementary material for: Systemic glucocorticoid exposure and postoperative infection risk in 143,782 appendectomy patients—a Danish longitudinal nationwide study
Source: Langenbecks Arch Surg. 2024 Mar 27;409(1):105. doi: 10.1007/s00423-024-03294-z (PMC10973007; doi:10.1007/s00423-024-03294-z)
Supplement: Supplementary file 1 — Supplementary file1 (DOCX 860 KB) [file 423_2024_3294_MOESM1_ESM.docx]

**Supplementary Materials**

**Title:**

Systemic glucocorticoid exposure and postoperative infection risk in 143,782 appendectomy patients - A Danish Longitudinal Nationwide Study

**Authors:**

Doruk Orgun, M.D., Ask Tybjærg Nordestgaard, M.D., Ph.D., Henrik Enghusen Poulsen, M.D., D.M.Sci., Ismail Gogenur M.D., D.M.Sci., Christina Ellervik M.D., D.M.Sci.

**Index:**

| **Online-only Figures and Tables** |  |
| --- | --- |
| Supplementary Table 1 | *Page 2* |
| Supplementary Table 2 | *Page 3* |
| Supplementary Table 3 | *Page 4* |
| Supplementary Figure 1 | *Page 5* |
| Supplementary Figure 2 | *Page 6* |
| Supplementary Figure 3 | *Page 7* |
| Supplementary Figure 4 | *Page 8-9* |

**Supplementary Table 1: Baseline characteristics of patients undergoing appendectomy in Denmark from 1996-2018 according to glucocorticoid exposure.**

|  | No/low glucocorticoid exposure*  (n = 142 698) | High glucocorticoid exposure*  (n = 1084) | Total  (n = 143 782) |
| --- | --- | --- | --- |
| Age, years (IQR**) | 29 (16-49) | 64 (42-74) | 29 (17-49) |
| Sex, n (%) |  |  |  |
| Female | 73 889 (51.8) | 654 (60.3) | 74 543 (51.8) |
| Length of stay, days (IQR**) | 2 (1-4) | 4 (2-7) | 2 (1-4) |
| Type of surgery, n (%) |  |  |  |
| Open  Laparoscopic | 76 266 (53.4)  66 432 (46.6) | 647 (59.7)  437 (40.3) | 76 913 (53.5)  66 869 (46.5) |
| Complicated appendicitis, n (%) | 35 039 (24.6) | 237 (21.9) | 35 276 (24.5) |
| Comorbidities, n (%) |  |  |  |
| Diabetes  Malignancy  Autoimmune disease  Inflammatory bowel disease  Obstructive pulmonary disease | 2914 (2.0)  8877 (6.2)  3933 (2.8)  1671 (1.2)  8489 (5.9) | 79 (7.3)  205 (18.9)  318 (29.3)  105 (9.7)  297 (27.4) | 2993 (2.1)  9082 (6.3)  4251 (3.0)  1776 (1.2)  8786 (6.1) |

* High glucocorticoid exposure: ≥5 mg prednisone equivalents per day, no/low glucocorticoid exposure: <5 mg prednisone equivalents per day.

** IQR: interquartile range

**Supplementary Table 2: Distribution of infection types between postoperative days 0-15 and postoperative days 16-90.**

|  | No/low glucocorticoid exposure | High glucocorticoid exposure | All patients |
| --- | --- | --- | --- |
| Infection type, n (%) | **Postoperative days 0-15** | | |
| SSI & Intraabdominal  Pneumonia & Sepsis  UTI and other | 2612 (31.7)  626 (7.6)  2701 (32.7) | 24 (18.9)  17 (13.3)  20 (15.7) | 2636 (31.5)  643 (7.7)  2721 (32.5) |
| *Subtotal* | (72.0) | (48.0) | (71.6) |
|  | **Postoperative days 16-90** | | |
| SSI & Intraabdominal  Pneumonia & Sepsis  UTI and other | 799 (9.7)  445 (5.4)  1067 (12.9) | 16 (12.6)  29 (22.8)  21 (16.5) | 815 (9.7)  474 (5.7)  1088 (13.0) |
| *Subtotal* | (28.0) | (52.0) | (28.4) |

*No/low glucocorticoid exposure: <5 mg prednisone equivalents per day, high glucocorticoid exposure: ≥5 mg prednisone equivalents per day, SSI: surgical site infection, UTI: urinary tract infection.

**Supplementary Table 3: Hazard ratio estimates for 90-day postoperative infections with high glucocorticoid exposure (≥5 mg prednisone equivalents per day) with no/low glucocorticoid exposure (<5 mg prednisone equivalents per day) as baseline with sensitivity analyses.**

| Method of sensitivity analysis  (in comparison with the Multivariate Cox regression model used in the primary analyses) | Adjusted hazard ratio  (95% confidence interval) | P value |
| --- | --- | --- |
| Without excluding patients with an infection diagnosis on the day of admission | 1.26 (1.03 – 1.53) | 0.02 |
| With mixed effect Cox regression to account for the clustering of patients at hospital level | 1.25 (1.03 – 1.52) | 0.03 |
| By the stratification of major disease groups where glucocorticoids are indicated | 1.23 (1.01 - 1-50) | 0.04 |
| By excluding the “complicated appendicitis” co-variable from the regression models | 1.25 (1.02 - 1.52) | 0.03 |


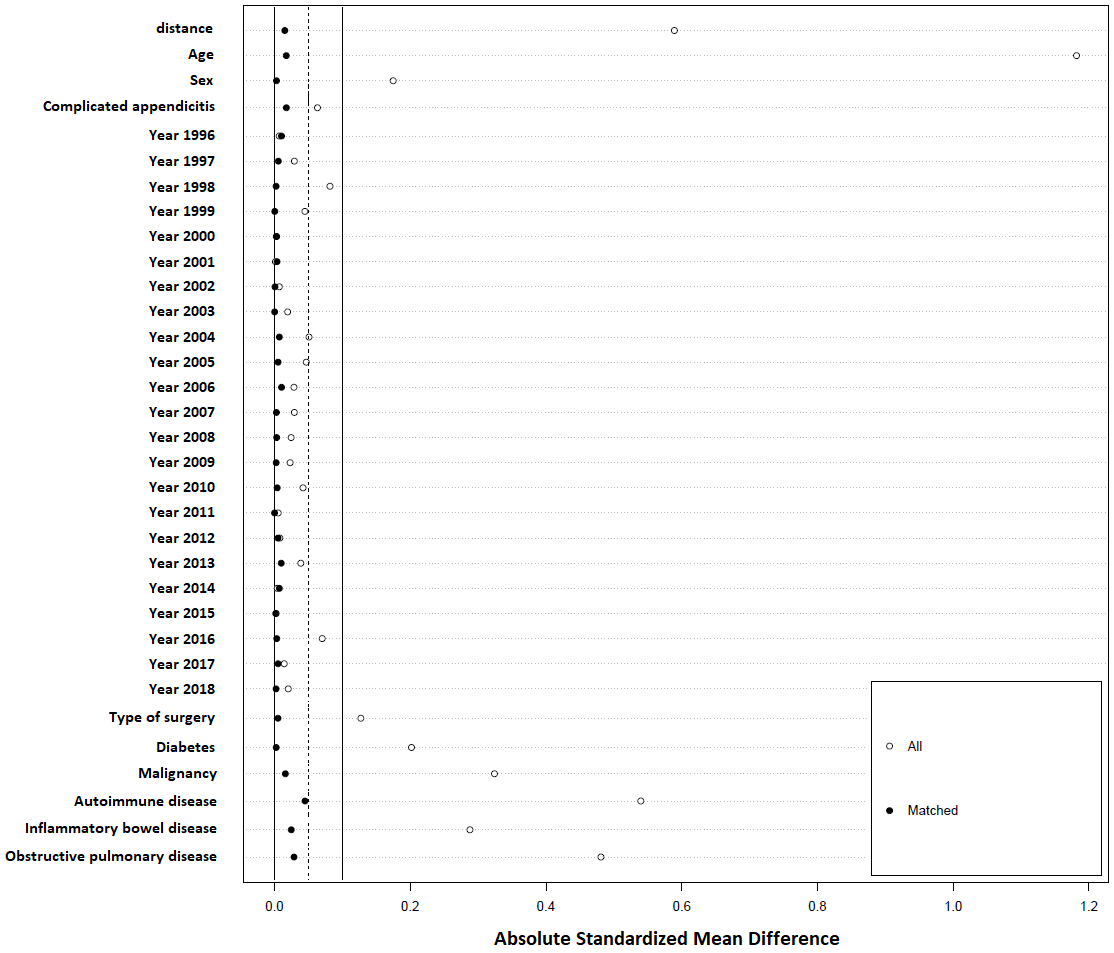


**Supplementary Figure 1:** Absolute standardized mean differences of all covariates before and after propensity score matching in patients undergoing appendectomy in Denmark from 1996-2018. High glucocorticoid exposure: ≥5 mg prednisone equivalents per day, low glucocorticoid exposure: >0 mg and <5 mg prednisone equivalents per day. For all co-variables, values less than 0.05 were achieved indicating acceptable covariate balance.


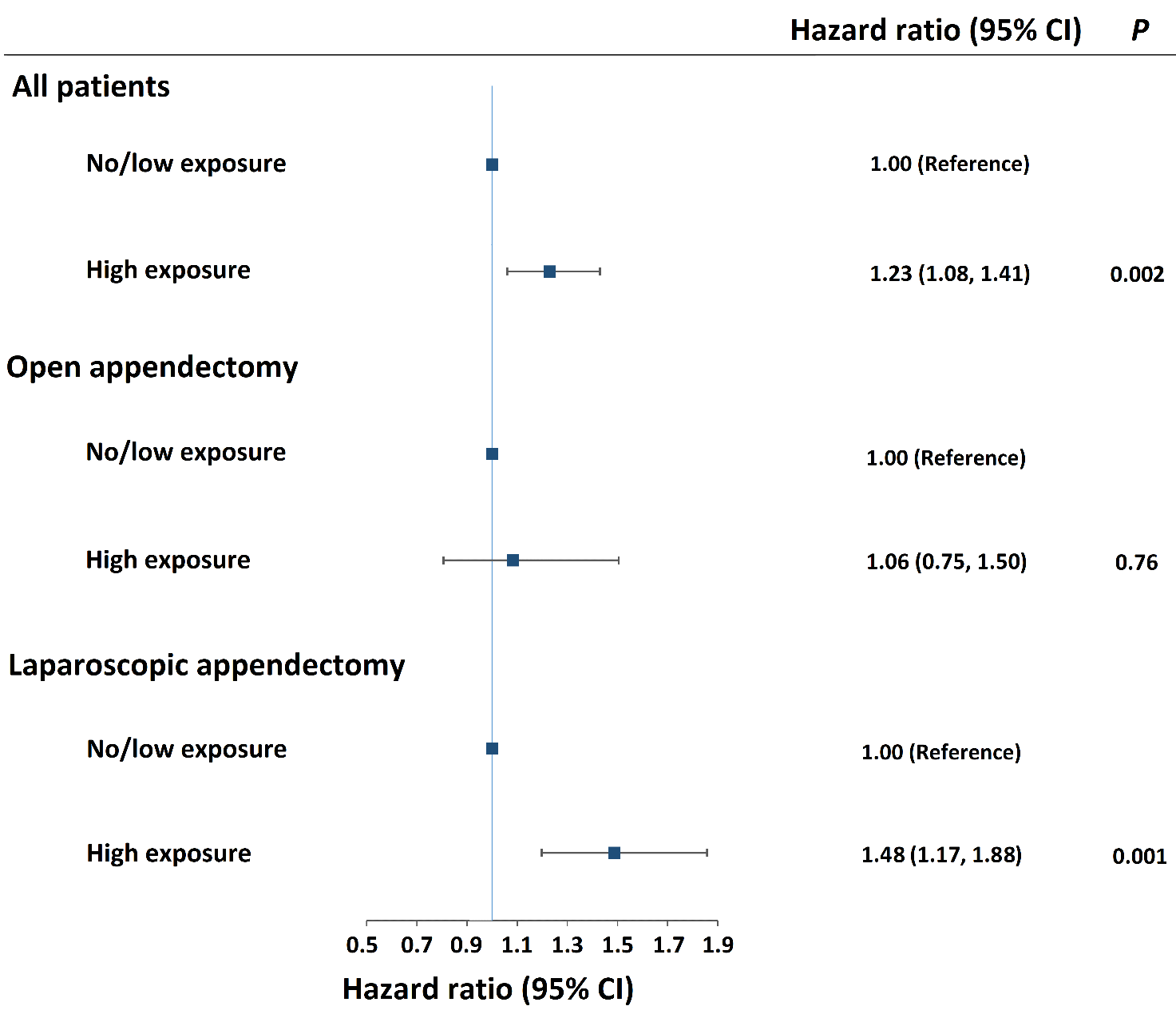


**Supplementary Figure 2:** Hazard ratios following propensity-score subclassification matching for postoperative infections within 90 days postoperatively for no/low glucocorticoid exposure (<5 mg prednisone equivalents per day) versus high glucocorticoid exposure (≥5 mg prednisone equivalents per day) in all patients, in open appendectomies, and in laparoscopic appendectomies. Multivariate Cox proportional hazards regression was performed by including the weights from the propensity-score matching. Point estimates and bars represent hazard ratios and 95% confidence intervals. Results show a similar increase in risk that was previously demonstrated with conventional covariate adjustment in Figure 3. CI = Confidence interval.

**
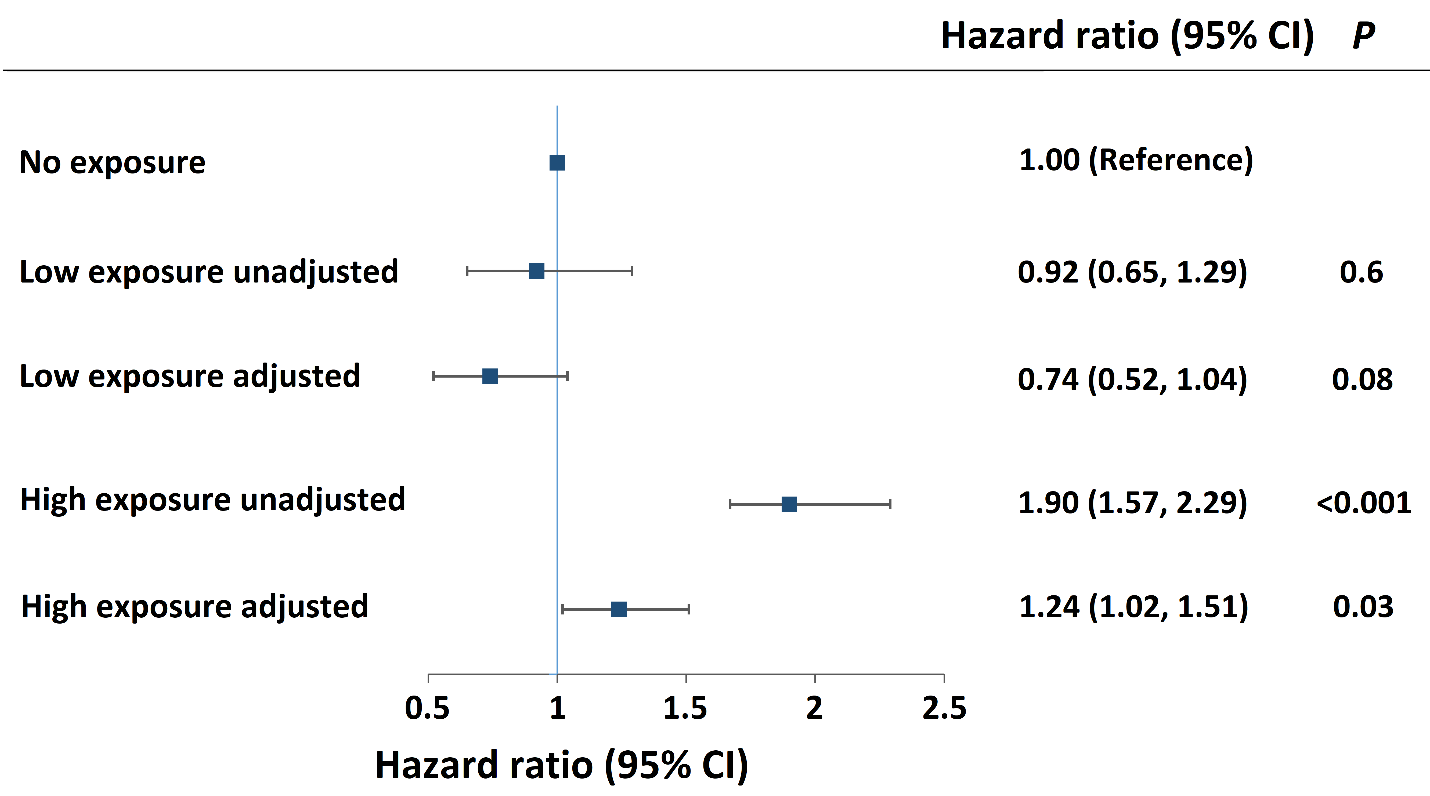
Supplementary Figure 3:** Hazard ratios for 90-day postoperative infections for no glucocorticoid exposure versus low glucocorticoid exposure (>0 mg and <5 mg prednisone equivalents per day) and high glucocorticoid exposure (≥5 mg prednisone equivalents per day) in all appendectomies. Hazard ratios according to Cox proportional hazards regression both before and after covariate adjustment are displayed. Point estimates and bars represent hazard ratios and 95% confidence intervals. CI = Confidence interval.


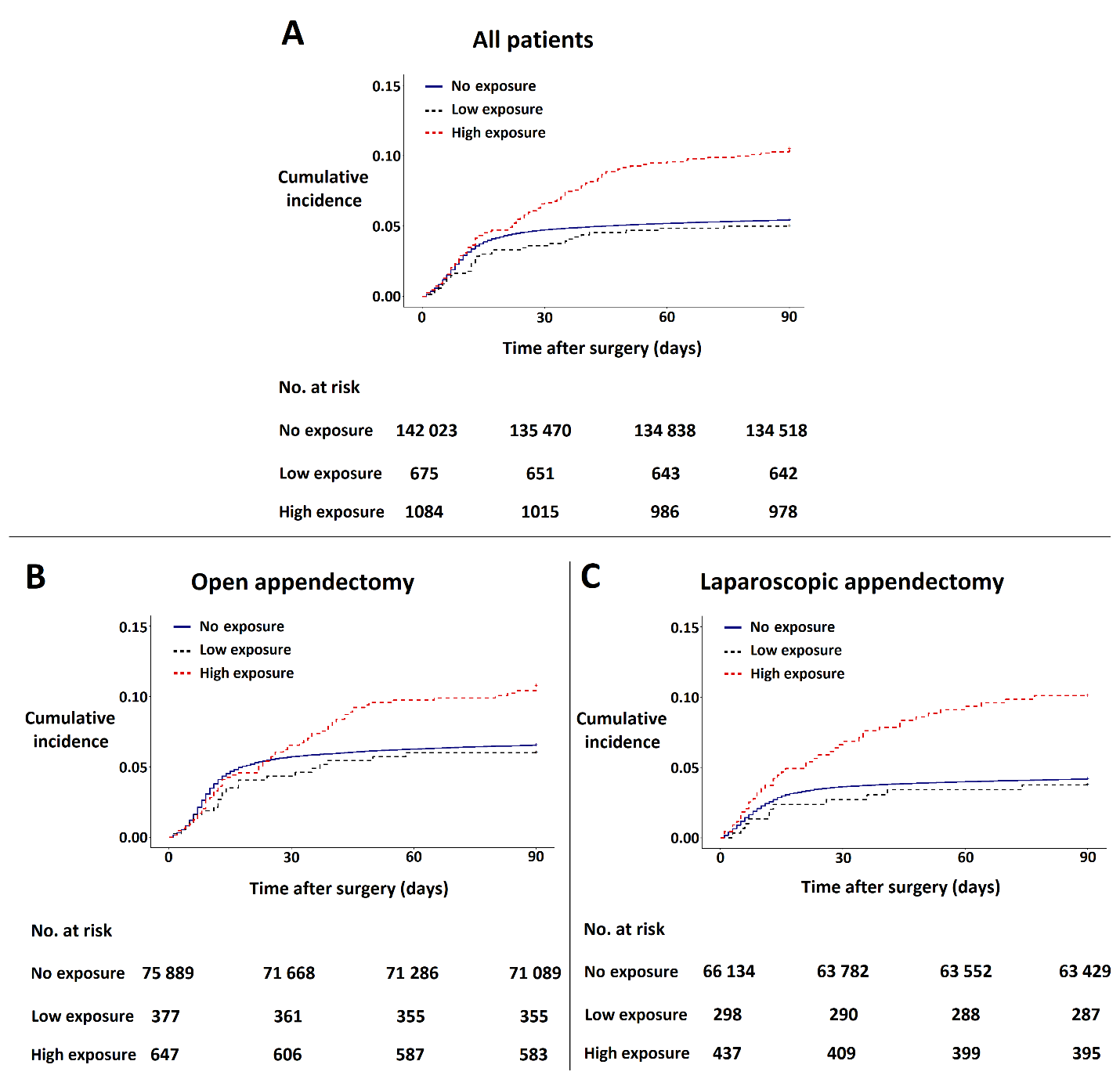


**Supplementary Figure 4:** Kaplan-Meier curves for the 90-day cumulative incidence of postoperative infections in patients undergoing appendectomy in all patients (A), in open appendectomies (B), and in laparoscopic appendectomies (C). Patients were stratified into groups of high (≥5 mg prednisone equivalents per day), low (>0 mg and <5 mg prednisone equivalents per day), and no glucocorticoid exposure. Log rank test P values for the comparison of no exposure with low exposure were 0.63 in all patients (A), 0.68 in open appendectomies (B), and 0.72 in laparoscopic appendectomies (C). Log rank test P values for the comparison of no exposure with high exposure were < 0.001 in all patients (A), open appendectomies (B), and laparoscopic appendectomies (C).
